# Evaluation of evidence grades in psychiatry and psychotherapy guidelines

**DOI:** 10.1186/s12888-020-02897-2

**Published:** 2020-10-12

**Authors:** Lisa Löhrs, Mirjam Handrack, Ina Kopp, Frank Jessen, Elias Wagner, Peter Falkai, Astrid Röh, Wolfgang Strube, Alkomiet Hasan

**Affiliations:** 1 Department of Psychiatry and Psychotherapy, University Hospital Munich, Ludwig-Maximilians Universität München, Nußbaumstraße 7, 80336 München, Germany; 2 Arbeitsgemeinschaft der Wissenschaftlichen Medizinischen Fachgesellschaften, Institut für Medizinisches Wissensmanagement, c/o Philipps-Universität Marburg, Marburg, Germany; 3 Department of Psychiatry, University of Cologne, Medical Faculty, Cologne, Germany; 4 Department of Psychiatry, Psychotherapy and Psychosomatics, Bezirkskrankenhaus Augsburg, University of Augsburg, Dr.-Mack-Straße 1, 86156 Augsburg, Germany

**Keywords:** German guidelines of psychiatry and psychotherapy, SIGN guidelines, Rrecommendations, Evidence grade, Distribution of recommendations

## Abstract

**Background:**

Information regarding the distribution of evidence grades in psychiatry and psychotherapy guidelines is lacking. Based on the German evidence- and consensus- based (S3) psychiatry and psychotherapy and the Scottish Intercollegiate Guidelines Network (SIGN) treatment guidelines, we aimed to specify how guideline recommendations are composed and to what extent recommendations are evidence-based.

**Methods:**

Data was collected from all published evidence- and consensus-based S3-classified psychiatry and psychotherapy guidelines. As control conditions, data from German neurology S3-classified guidelines as well as data from recent SIGN guidelines of mental health were extracted. Two investigators reviewed the selected guidelines independently, extracted and analysed the numbers and levels of recommendations.

**Results:**

On average, 45.1% of all recommendations are not based on strong scientific evidence in German guidelines of psychiatry and psychotherapy. A related pattern can be confirmed for SIGN guidelines, where the mean average of recommendations with lacking evidence is 33.9%. By contrast, in the German guidelines of neurology the average of such recommendations is 16.5%. A total of 24.5% of all recommendations in the guidelines of psychiatry and psychotherapy are classified as level A recommendations, compared to 31.6% in the field of neurology and 31.1% in the SIGN guidelines. Related patterns were observed for B and 0 level recommendations.

**Conclusion:**

Guidelines should be practical tools to simplify the decision-making process based on scientific evidence. Up to 45% of all recommendations in the investigated guidelines of psychiatry and psychotherapy are not based on strong scientific evidence. The reasons for this high number remain unclear. Possibly, only a limited number of studies answer clinically relevant questions. Our findings thereby question whether guidelines should include non-evidence-based recommendations to be methodologically stringent and whether specific processes to develop expert-opinion statements must be implemented.

## Background

Evidence-based medicine aims to integrate the best research evidence with clinical expertise and patient values [1]. In this regard, guidelines are systematically developed tools to assist physicians and other health-care professionals in the decision-making process and to support the transparency of medical decisions. Apart from summarising the current medical evidence, guidelines are instruments to weigh the risk-benefit ratios of diagnostic procedures and treatments. Guidelines are also instruments of quality management of the healthcare system and viewed to improve quality and effectiveness of clinical as well as costs of healthcare [2].

In Germany, the Association of the Scientific Medical Societies (AWMF) started coordinating the development of guidelines in 1995. Since 1997 guidelines are cost-free and publicly available online [3]. In contrast to other countries, clinical guidelines in Germany are not state-funded. For example, in the UK the National Institute of Clinical Excellence (NICE) orders the development of a clinical guideline from a group of independent experts and finances the process [4]. In Germany, the development is only financed by the subscriptions of the publishing medical societies [3] and by the cost-free work of the people involved. Because of the costly and time-intensive development process of high-quality clinical guidelines, different options for funding are currently in discussion. The objective would be an independent and sustainable financial support [5] to accelerate the development process, to consider the most recent study results and to evaluate the guidelines. Despite an important legislative initiative to improve the funding situation [6], it is not foreseeable that there will be a fundamental change regarding the way how guidelines are developed in Germany.

Regardless of any financial consideration, the development process of German guidelines follows standard operation procedures, developed by the AWMF aiming at providing the same level of quality across guidelines. More than 20 years ago clinical guidelines in Germany were not subject to standardisation, so the AWMF started an extensive program in 1998 to improve their quality [3]. For this purpose, the German Rating Instrument for Guideline Valuation (DELBI) has been developed based on the Appraisal of the Guidelines for Research and Evaluation instrument (AGREE, DELBI domains 1 to 6) and was adapted for the German healthcare system (DELBI domain 7) to minimise differences concerning quality and underlying methodology across guidelines and to ensure inter-comparability [7]. Similar efforts were made by the Scottish Intercollegiate Guidelines Network (SIGN) as summarised in the SIGN guidelines developer’s handbook.

In that regard, three major classifications for guidelines can be distinguished in Germany. Groups of experts develop in an informal consensus (‘expert recommendations’), which results in so called S1-classified guidelines. By comparison S2-classified guidelines need a formal multidisciplinary consensus. Since 2004, the differentiation between S2e-classified (‘evidence-based’) and S2k-classified (‘consensus-based’) exists. S2k-guidelines are not evidence-based and recommendations are not graded, but the consensus process is standardised and has to follow standard operating procedures. For the development of S2e-guidelines, a systematic research process and the selection and evaluation of evidence are required. S3-classified guidelines are evidence- and consensus-based and have the highest methodological quality as well as the most expensive development process. After a structured literature research and rating of the evidence, they have to fulfil different DELBI criteria [3]. AWMF guidelines use either the Oxford Centre for Evidence Based Medicine (OCEBM) or the SIGN grading systems and AWMF S3-guidelines underlie a structured consensus process which finally leads to the recommendations (grades A, B, 0 and CCP, see Table 1). AWMF was one of the funding organisations of the Guidelines International network and from the international perspective, AWMF S3-guidelines are rooted in one of the most elaborated development processes.

**Table 1:** Grade of recommendation and underlying levels of evidence (SIGN and OCEBM); following the SIGN and the OCEBM grading system the levels of evidence are rated in four or five categories. Results of studies can be extrapolated or interpolated so that lower levels of evidence can underlie higher grades of recommendations and conversely

Grade of recommendation	Description	Level of evidence (SIGN)	Level of evidence (OCEBM)
**A**	Strong recommendation	1++, 1+, (1-)	I, (II)
**B**	Recommendation	2++, 2+, (2-)	II, III, (I)
		or downgrading of 1++ / 1+ / (1-) due to methodological considerations	
**0**	Open recommendation	3,4	IV, V, (II, III)
		or downgrading of 2++ / 2+ / (2-) due to methodological considerations	
**CCP**	Clinical consensus (different strengths of recommendation possible)	No level of evidence	No level of evidence

Since 1998 the total number of published medical guidelines in Germany has been growing continuously, but over the last years the proportion of S1 to S3 guidelines has changed. In 2002, 445 S1-guidelines, 121 S2-guidelines and 17 S3-guidelines were available. In 2016, 108 S3-guidelines were available and 374 guidelines were newly registered [8]. Since 2014 the number of newly registered guidelines has decreased, but with a continuously increasing number of high-quality guidelines [8, 9] which might confirm that the process of improving the quality of guidelines in general has been successful so far.

High methodological quality is essential to guarantee recommendations based on scientific evidence with practical relevance and to minimise possible conflict of interest biases. Moreover, the amount of recommendations and their respective grading levels are important from the view of usability. However, the way of how to grade evidence and to develop guidelines differs between countries. Nonetheless, most guidelines use the original or the adapted version of the Grading of Recommendations Assessment, Development and Evaluation (GRADE) process [10].

Despite important efforts to harmonise and improve the guideline development processes, guidelines do have a relevant proportion of non-evidence-based recommendations that are presumably expert opinions. In 2014 one study of the Scottish Intercollegiate Guidelines Network (SIGN) (somatic disciplines and mental health) evaluated the 42 national guidelines with respect to the quantity and quality of the given recommendations. The authors observed that only 24% of all recommendations were based on high scientific evidence (denoted as level A recommendations). Remarkably, more than a third of all recommendations were expert-based recommendations and more than a half of the recommendations were based on limited scientific evidence [11]. Moreover, these results highlight that the number of recommendations with low or absent evidence increases with the total amount of recommendations.

The challenge to develop high-quality guidelines in psychiatry and psychotherapy is all the greater compared to somatic disciplines due to the implementation of patient reported outcomes. So called “soft endpoints”, which are composed of patient and observer reported outcomes collected via self- or observer-rated clinical rating scales, questionnaires, or interviews [12]. Moreover, psychiatric disorders are subject to a high complexity of diagnosis, treatment, extensive involvement of patients and relatives and outcome measurements. One could speculate that the desire of the guideline developers to deal with this high complexity could possibly lead to a high amount of not evidence-based recommendations. To test this hypothesis, we conducted an analysis of distribution of recommendation grades in evidence- and consensus-based German guidelines of psychiatry and psychotherapy compared to national guidelines of neurology and to the internationally established SIGN guidelines.

## Methods

This study focuses on all available German S3-guidelines in psychiatry and psychotherapy in their respective most recent versions (status July 2019). The S3-guidelines of neurology were investigated to serve as a first control group to allow a comparison to a medical field with an overlap in diagnoses or treatments. The recent SIGN guidelines of mental health (status July 2019) were investigated as second control group taking into account the study from Baird and colleagues published in 2014 [11]. Moreover, SIGN guidelines are based on related development procedures to German S3-guidelines in evaluating and grading evidence allowing for a direct comparison. Indeed, there are differences between the SIGN- and the German S3- guideline developing process. The most important difference is the composition of the guideline developing group: the SIGN Executive discusses which specialties and professions should be represented on the guideline development group with the topic proposer(s). This process is advised from the appropriate specialty subgroup(s) and SIGN Council. SIGN guideline development groups vary in size depending on the scope of the topic under consideration, but generally comprise between 15 and 25 members. In contrast, the AWMF only proposes that all relevant interest groups should be represented, without recommendations about the size of the developing group. The respective medical societies are responsible for assembling the guideline group and developing the guideline. The AWMF provides the methodological framework for developing the guidelines and serves as the publisher.

To identify guidelines of interest, we screened the AWMF database, the webpages of the publishing medical societies and the SIGN webpage for all guidelines covering psychiatric or neurological disorders. Two independent investigators (MH and LL) selected and evaluated all AWMF S3-guidelines published by the German Society for Psychiatry and Psychotherapy (DGPPN) and the German association for Neurology (DGN) and the respective SIGN guidelines. If investigators had different results, these results were discussed with a third investigator (AH) until a final consensus-decision was reached.

**Fig. 1:**
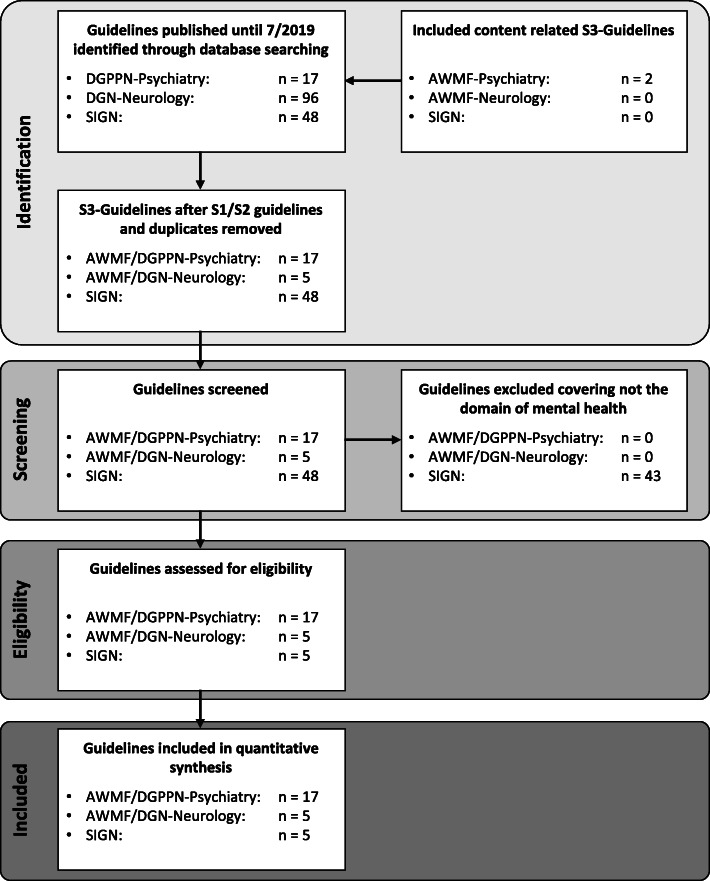
PRISMA flowchart

15 from 17 guidelines, published by the DGPPN were S3-guidelines. The two excluded guidelines were guidelines classified as S1 and S2: the S1-guideline for disorders of sexual preferences and the S2-guideline for personality disorders. We included the two content related S3-guidelines “Functional body complaints” and “Post-traumatic stress disorder”, published only by the AWMF, resulting in a total of 17 S3-guidelines of psychiatry and psychotherapy. The DGN published a total of 96 guidelines with only five S3-guidelines. Apart from recent guidelines (*n* = 18), we included expired guidelines and guidelines under review as well (*n* = 4). In contrast to the SIGN guidelines, where expired guidelines are automatically removed after 10 years, the German guidelines are still available and used in clinical practice years after their expiration (e.g. see guideline “Obsessive compulsive disorder”, which expired 2018 and is now “under revision”). The dementia guideline as a joint publication of DGPPN and DGN was listed twice but was analysed as psychiatric guideline. 5 of 48 currently available SIGN guidelines were guidelines covering the domain of mental health. Taking the information of the SIGN 50 developer’s handbook into consideration, these five guidelines were methodologically comparable to German S3-guidelines [13–17]. Please see Fig. 1 and Table 1 for more information reading the selection process.

Guidelines were analysed regarding the amount of recommendations, the distribution of recommendation grades, the size of the responsible consensus groups (the latter only applicable to German S3-guidelines). An analysis of the size of the SIGN consensus groups was not performed due to the mentioned differences in the development process and in the composition of these groups. Since guidelines sometimes applied different wording for grading non-evidence-based recommendations, we summarised those recommendations as “clinical consensus points” (CCP). This label included the terms “*Klinischer Konsensus Punkt*” (clinical consensus point), “*Expertenkonsens*” (expert consensus) and “*GCP*” (Good Clinical Practice). The recommendation levels A, B and 0 were consistently used in all analysed guidelines (also referring to Table 1 for more detailed information on the grading system). However, the term “statement” was used for all recommendations or declarations especially mentioned and highlighted in the guidelines without having a level of evidence or grading of recommendations. In contrast to A, B, 0 or CCP recommendations statements neither involve a recommendation grade nor are they based on scientific evidence. Nevertheless, they are based on an expert consensus and can be seen as additional material to the recommendations. Statements were counted separately and not included in the statistics. In some guidelines, recommendations with two gradings like ‘A/CCP’ were available. In such cases, both recommendation categories were taken into account for further analyses.

### Statistics

We based all analyses on the long versions of the guidelines. The rational for using the long version was, that we initially expected that the long and short versions would include the same number of recommendations. However, as detailed below, we detected inconsistencies regarding the number of recommendations in seven of the German S3-guidelines. All analyses were computed using IBM SPSS 25 (IBM, Armonk, NY, USA) with a significance level of α = 0.05. Descriptive statistics include means, standard deviations and maximum and minimum counts. Mean relative frequencies of the four recommendation grades (A, B, 0, CCP) for each of the three study groups separately (AWMF psychiatry and psychotherapy, AWMF neurology, SIGN mental health) were calculated based on the individual relative frequencies of recommendation grades within each guideline (absolute count of a given recommendation grade (e.g. A or CCP)/absolute total count of all recommendations). Explorative Pearson correlation analyses were conducted to investigate correlations between the size of the consensus group and the relative frequencies of a given recommendation grade and between the amount of CCP recommendations and the total of recommendations in German S3-guidelines.

## Results

In July 2019, 18 out of the 22 German guidelines were available as a recent or updated version: 14 of them were guidelines of psychiatry and psychotherapy and four guidelines of neurology. The four remaining guidelines were under revision. 5 of the available and current 48 SIGN guidelines were guidelines covering the domain of mental health. For 14 of the German S3-guidelines a short version was available. In 7 out of 11 [18–24] of the guidelines of psychiatry and psychotherapy and in three out of three neurology guidelines [25–27] we detected discrepancies in the amount of recommendations between long and short versions. Four of the German guidelines of psychiatry and psychotherapy and one neurology guideline are based on the SIGN grading system. The remaining guidelines used the OCEBM system to grade evidence.

In the AWMF psychiatry and psychotherapy guidelines, 354 level A recommendations with a mean relative frequency of 24.5% (± 19.3%; max: 76.5%, min: 0%) and a mean absolute frequency of 20.8 (± 18.9; max: 77, min: 0) could be detected. In contrast, the AWMF guidelines of neurology had a total of 76 level A recommendations with a mean relative frequency of 31.6% (± 21.6%; max: 65.8%, min: 8%) and a mean absolute frequency of 15.2 (± 8.8, max: 25, min: 2). The SIGN guidelines of mental health had a total of 46 level A recommendations with a mean relative frequency of 31.1% (± 15%; max: 55.6%, min: 19.6%) and a mean absolute frequency of 9.2 (± 5.8; max: 19, min: 5).

The mean relative frequency of level B recommendations of the AWMF guidelines of psychiatry and psychotherapy was 18.5% (± 10.8%; max: 40.4%, min: 0%) with a total of 320 (18.8 ± 14.3; max: 44, min: 0) level B recommendations. In the AWMF neurology guidelines, the mean relative frequency was 29.4% (± 14.5%; max: 52%, min: 16.7%) based on a total of 88 recommendations (13.6 ± 6.8; max: 21, min: 6). In the SIGN guidelines, a total of 45 level B recommendations with a mean relative frequency of 29% (± 13.2%; max: 44.4%, min: 12.4%) and an absolute mean frequency of 9 (± 6.5; max: 19, min: 4) were counted.

A related pattern could be observed for level 0 recommendations, with a total of 210, a mean relative frequency of 11.8% (± 8.7%; max: 30.8%, min: 0%) and a mean absolute frequency of 12.4 (± 12; max: 48, min: 0) in the AWMF guidelines of psychiatry and psychotherapy. In the guidelines of neurology the mean relative frequency of level 0 recommendations was 22.5% (± 15.4%, max: 40%, min: 5.6%), with a total of 54 (10.8 ± 7.8; max: 21, min: 2), while the SIGN guidelines had a mean relative frequency of 6% (± 7.2%; max: 17.6%, min: 0%) with a total of 12 (2.4 ± 2.5; max: 6, min: 0).

The AWMF guidelines of psychiatry and psychotherapy had a higher relative mean frequency (45.1% ± 26.6%; max: 100%, min: 0%) of CCP recommendations based on a total number of 752 (44.2 ± 32.8, max: 109, min: 0) compared to the AWMF guidelines of neurology with a relative mean frequency of 16.5% (± 22.6%; max: 41.7%, min: 0%), based on a total of 55 (11 ± 17.5; max: 40, min: 0) of CCP recommendations. The SIGN guidelines had a mean relative frequency of 33.9% (± 24.3%; max: 61.9%, min: 0%) based on a total of 92 CCP recommendations (18.4 ± 24.1; max: 60, min: 0) (see Tables 2 to 3).

**Table 2:** Total amount of A, B, O and CCP recommendations as well as statements and size of the consensus group for each guideline. Year of publication and grading system are also specified for each guideline

	A	B	0	CCP	Statements	Total without statements	Size of consensus group
**AWMF guidelines of Psychiatry and Psychotherapy total (year of publication/Grading system)**	354	320	210	752	184	1636	
Attention deficit hyperactivity disorder (2018/SIGN)	7	16	4	57	0	84	27
Schizophrenia (2019/SIGN)	32	30	9	99	8	170	38
Dementia (2016/OCEBM)	12	38	24	20	3	94	25
Obsessive compulsive disorder (2013/OCEBM)	11	8	10	34	7	63	41
Bipolar Disorder (2019/SIGN)	6	36	48	66	77	156	19
Psycho-social therapy in severe mental illness (2019/OCEBM)	10	8	0	15	11	33	42
Methamphetamine related disorders (2016/OCEBM)	77	35	17	0	7	129	17
Unipolar Depression (2015/OCEBM)	33	44	24	31	15	132	28
Screening, diagnosis and treatment of alcohol related disorders (2016/OCEBM)	48	28	15	92	0	183	47
Screening, diagnosis and treatment of tobacco related disorders (2015/OCEBM)	30	21	12	15	0	78	48
Treatment of anxiety disorders (2014/OCEBM)	17	7	3	34	0	61	37
Diagnosis and treatment of eating disorders (2010/OCEBM)	11	13	14	64	1	102	24
Psycho-oncological diagnostics, advising and treatment of adult cancer patients (2014/OCEBM)	9	0	1	43	1	53	52
Post-traumatic stress disorder (2011/OCEBM)	13	2	2	0	0	17	20
Definition, pathophysiology and diagnosis of Fibromyalgia (2017/OCEBM)	20	24	10	34	48	88	35
Functional body complaints (2018/OCEBM)	0	0	0	109		109	32
Prevention of coercive interventions: Prevention and treatment of aggressive behavior in adults (2018/OCEBM)	18	10	17	39	6	84	25
**AWMF guidelines of Neurology total**	76	68	54	55	40	253	
Diagnosis and treatment of the cubital tunnel syndrome (2017/OCEBM)	2	13	10	0	9	25	7
Axonal injury, medical care of peripheral axonal injuries (2013/OCEBM)	15	20	21	0	16	56	10
Parkinson (2016/SIGN)	21	21	16	40	0	98	30
Lyme neuroborreliosis (2018/OCEBM)	25	8	5	0	0	38	15
Secondary prophylaxis of ischemic stroke and transient ischemic attack (2015/OCEBM)	13	6	2	15	15	36	19
**SIGN guidelines of mental health total**	46	45	12	92		195	
Risk reduction and management of delirium (2019/SIGN)	6	6	0	13		25	
Assessment, diagnosis and interventions for autism (2016/SIGN)	19	12	0	60		97	
Management of schizophrenia (2013/SIGN)	10	19	3	15		47	
Management of attention deficit and hyperkinetic disorders in children and young people (2009/SIGN)	6	4	3	4		17	
Non-pharmaceutical management of depression (2010/SIGN)	5	4	0	0		9

**Table 3:** Statistical analysis of mean, standard deviation (SD), minimum and maximum of the relative and absolute amount of recommendations. Psychiatry refers to psychiatry and psychotherapy

	N	Mean [% (absolute)]	SD [% (absolute)]	Minimum [% (absolute)]	Maximum [% (absolute)]
AWMF Psychiatry	17				
A		24.5 (20.8)	19.3 (18.9)	0 (0)	76.5 (77)
B		18.5 (18.8)	10.8 (14.3)	0 (0)	40.4 (44)
0		11.8 (12.4)	8.7 (12.0)	0 (0)	30.8 (48)
CCP		45.1 (44.2)	26.6 (32.8)	0 (0)	100 (109)
AWMF Neurology	5				
A		31.6 (15.2)	21.6 (8.8)	8 (2)	65.8 (25)
B		29.4 (13.6)	14.5 (6.8)	16.7 (6)	52 (21)
0		22.5 (10.8)	15.4 (7.8)	5.6 (2)	40 (21)
CCP		16.5 (11)	22.6 (17.5)	0 (0)	41.7 (40)
SIGN	5				
A		31.1 (9.2)	15 (5.8)	19.6 (5)	55.6 (19)
B		29 (9)	13.2 (6.5)	12.4 (4)	44.4 (19)
0		6 (2.4)	7.2 (2.5)	0 (0)	17.6 (6)
CCP		33.9 (18.4)	24.3 (24.1)	0 (0)	61.9 (60)

Only five of the 22 German S3-guidelines, 2 from psychiatry and psychotherapy and 3 from neurology, had exclusively level A, B and 0 recommendations and no CCP recommendation [27–31]. However, the guideline of functional body complaints provided only CCP recommendations [23]. In the SIGN guidelines of mental health one out of five guidelines had no CCP recommendation (guideline on non-pharmaceutical management of depression).

Exploratory correlation analyses between the number CCP recommendations and the total amount of recommendations showed a statistically significant positive correlation (*r* = 0.625, *p* = 0.007) for the German guidelines of psychiatry and psychotherapy, but no statistically significant correlation for the neurology guidelines (*r* = 0.835, *p* = 0.078). For the SIGN guidelines of mental health, the analyses showed also a statistically significant positive correlation (*r* = 0.978, *p* = 0.004). Analysing the German S3 guidelines of psychiatry and neurology together, as well as all guidelines included in this study, the number of CCP recommendations correlated significantly with the total number of recommendations. (*r* = 0.706, *p* < 0.001; *r* = 0.745, *p* < 0.001).

For the AWMF guidelines of psychiatry and psychotherapy only the exploratory correlation analyses between the size of the consensus group and the relative frequencies of grade 0 recommendations showed a statistically significant negative correlation (*r* = − 0.516; *p* = 0.034). All other correlation analyses for both AWMF groups separately, did not show significant correlations between the relative frequency of a given recommendation grade and the size of the guideline group. Analysing all AWMF guidelines (*N* = 22), a statistically significant negative correlation for grades B (*r* = − 0.498 and *p* = 0.018) and 0 (*r* = − 0.632, *p* = 0.002) and a statistically positive correlation for CCP recommendations (*r* = 0.591; *p* = 0.004) with the size of the guideline group were detected. These correlations indicate that in guidelines with increasing size of the consensus groups, the number of CCP recommendations increases as well. For B and 0 recommendations, the reverse relationship was observed. For grade A recommendations, no significant correlation was detected (*r* = − 0.176; *p* = 0.435).

## Conclusion

Our study shows for the first time that the relative mean frequency of recommendations in the German psychiatry and psychotherapy guidelines, which are not rooted in strong scientific evidence amounts to 45.1%. In neurology guidelines this frequency is only 16.5%. This observation of a high frequency of CCP recommendations in the German psychiatry and psychotherapy guidelines is even higher than in the SIGN guidelines of mental health with a relative mean frequency of 33.9%. Independent of the discipline, the relative mean frequency of CCP recommendations correlates positively with the size of the consensus group in the evaluated German guidelines. In general, an increasing number of recommendations correlates positively with a higher number of CCP recommendations.

The reasons for these phenomena remain elusive, but one could build several hypotheses to explain the high proportion of CCP recommendations in psychiatry and psychotherapy guidelines. First, one could assume that for some clinically relevant questions respective clinical trials and subsequent meta-analyses are yet lacking. In this regard, one might argue that the responsible guideline groups aim to ask and try to answer such questions that cannot be addressed ethically in clinical trials but are of particular importance for clinical practice. Such scenarios include e.g. suicidality, coercive treatment or the need of supervision as part of the therapeutic process. Being aware that we only investigated two different medical disciplines, one could speculate, that the differences in the numbers of evidence- and consensus-based guidelines between psychiatry/psychotherapy and neurology (only five of 96 were classified as an S3-guideline) could indicate that in neurology the responsible society tries to avoid the development of such time- and money-consuming guidelines. Concerning the SIGN guidelines, the mean frequency of 33.9% of CCP recommendations shows that the observation of a high number of non-evidence-based recommendations in guidelines of psychiatry and psychotherapy seems to be an international phenomenon. Based on the additional analyses of the correlation between the total of recommendations and CCP recommendations, the findings of Baird et al. seem to affect only the German guidelines of psychiatry and psychotherapy. Analysing all SIGN guidelines Baird et al., detected a positive correlation between the increasing number of recommendations and the proportion of CCP recommendations. In our analyses we could confirm this relationship showing that this is an international phenomenon.

This overall effect and the limited scientific evidence in a magnitude of psychiatry and psychotherapy guidelines are critical, because such effects may support the self-confidence and interests of expert groups [32]. The impact of conflict of interest on guideline recommendations is evident and has been extensively investigated [33, 34]. A relevant risk of bias can be assumed based on our analyses of the number of recommendations in the S3-guidelines of psychiatry and psychotherapy and the SIGN guidelines. This conclusion cannot be generalized for those recommendations that were based on respective scientific evidence (mainly recommendations for pharmacological and psychotherapeutic interventions). One could speculate, that our exploratory finding of a positive correlation of the size of the consensus group and the relative frequency of CCP recommendations could support a possible relationship between financial and non-financial (e.g. membership in specific societies, affiliation to a specific therapeutic school, offering of specific courses) conflicts of interest and the approval of non-evidence-based recommendations. The greater the size of the consensus group, the more different groups of interest are represented. A high number of different stakeholders may possibly lead to an influence of the CCP recommendations by the different groups to maintain their own interests. However, we did not investigate the conflict of interest statements in the included guidelines, but taking into account earlier findings that in all German S3- guidelines only 76% contain a detailed declaration of the conflicts of interests and in total 55% of the participants of the guideline groups report any conflict of interest [32], one could speculate about a relationship between conflicts of interest and the amount of non-evidence-based recommendations. However, we could not establish this effect when both disciplines were analysed separately.

Additionally, it appears of importance to differentiate between “expert opinion” and “CCP” recommendation: “Expert opinion” describes a level of evidence, not a grade of recommendation. Even a CCP recommendation does not automatically mean, “lack of scientific evidence” nor it is only “eminence based” [35]. Though in the grading system the level of evidence on which CCP’s are based, is “no level of evidence”. This rather means, there is lack of literature research or the results of the investigated studies do not fully address the question of the guideline and have to be adjusted. For the most questions, studies or at least case reports exist. This points to some kind of a scientific background, which could be transferred to the questions of the guideline. One could speculate, that for recommendations where the guideline group has to transfer results, case reports or sometimes even practical knowledge in terms of “good clinical practice” room for interpretation is left. In these cases, the personal view of every member of a guideline group is more emphasized.

In this context, it may be possible that small groups of experts and stakeholders will drive the practice of a specialty in one direction. At the same time, respective high-quality clinical trials or meta-analyses in psychiatry and psychotherapy beyond studies regarding efficacy and effectiveness of pharmacological and psychotherapeutic interventions are lacking. In our view, there seems to be two possible options to address the outlined issue of conflicts of interest: The first option would be, not to provide any recommendations for clinical scenarios where no evidence is available yet. However and as discussed before, treatment guidelines without any non-evidence-based recommendations while having a higher methodological standard, would potentially ignore many clinically important scenarios and questions. The latter may result in a guideline that might be less useful in clinical practice due to the lack of importance and applicability for a variety of questions. Moreover, one should bear in mind that the absence of evidence does not equal the evidence of absence. For example, until today no randomised-controlled trial has been conducted to show that using a parachute is more effective to save lives than not using parachutes [36]. The second option would be to accept the aforementioned potential risk of bias and include recommendations based on expert consensus, as these can address clinically meaningful questions and scenarios. Obviously, such recommendations can be useful where clinical trials and meta-analyses are missing to facilitate the clinical decision-making process and to contextualise the decision-making of individuals with the views of experts. By contrast, a high number of such recommendations can foster uncertainty in the clinical decision-making process [37] due to the aforementioned increase in the risk of bias. Situations where recommendations based on expert consensus are necessary and helpful are not rare, neither in psychiatry and psychotherapy nor in other medical disciplines. Such areas are e.g. situations of ultra-treatment resistance, areas that are difficult to study (e.g. suicidality, coercive treatment), the management of rare side-effects, the combination treatment with different pharmacological compounds or the evaluation of long-term effectiveness and tolerability. In these cases, standard operating procedures (SOPs) on how to develop and approve non-evidence-based recommendations should be implemented for the guideline developing process. Such SOPs that provide a process for the development of CCP recommendations are missing in the AWMF and SIGN guideline developer’s handbooks. These SOPs could e.g. describe exactly the development and composition of each CCP recommendation and include the total number of CCP recommendations (e.g. not more than 15% or 20% of all recommendations) to force guideline developers to limit these recommendations to a minimal clinically relevant amount or the need to involve independent international experts in the process (e.g. using the Delphi method).

Another aspect is that the composition of the guideline-developing group could influence the amount of CCP recommendations. Following Baird and Lawrence [11], a high amount of recommendations based on expert opinions could be seen as a reflection how professional groups deal with uncertainty [11]. Moreover, they speculate [11] that expert groups may view their opinion as clinical experts being more authoritative than scientific evidence [38]. The more experts from different fields and interest groups are involved, the more differing opinions might exist and the more uncertainty might arise. Thus, the important development towards multi-professional guideline groups, involving stakeholders, relatives and other involved persons may foster the inflation of non-evidence-based recommendations. This may be especially true, as in many of the German S3-guidelines in psychiatry and psychotherapy involved groups the same persons are responsible for different guidelines (e.g. being a delegate for guidelines in general). Moreover, in contrast to e.g. SIGN, AWMF gives no restriction concerning the maximum size of the guideline group. Our general finding that the more groups are involved in a guideline, the more non-evidence-based recommendations are included supports to the described dilemma. Potential solutions could be to limit the size of the guideline groups, to limit the possibility for one person to participate in several guideline-development processes, to define standards of the qualifications of the involved person and to develop between-guideline standards for the here raised important issues.

Our analysis showed also that in some guidelines the number of recommendations differs between the short and the long version. Furthermore, in some guidelines the grading differed between both versions (e.g. in the guideline of Obsessive Compulsive Disorder recommendation number 4–10 is graded as CCP in the short version and as a statement in the long version [19]). Such findings question whether short versions of guidelines are necessary and might motivate guideline developers to pay attention to such discrepancies.

As a limitation, we only included guidelines of neurology as control group from Germany, which consisted only of five S3-guidelines. By comparison, analyses such as the one presented by Baird and Lawrence for the SIGN guidelines are not available for Germany. Thus, we cannot derive statements with respect to other medical disciplines. Moreover, with a total of 96 the number of neurology guidelines is very high and we assume that the only available five S3 guidelines may have had a stricter methodology approach than the remaining 91 resulting in an overestimation of the frequency of evidence-based recommendations. Secondly, our correlative findings are only significant when both disciplines are analysed. Another limitation is, that we solely analysed the current published SIGN guidelines of mental health for an international comparison, which consisted also only in five guidelines. Moreover, four of the German guidelines were out of date, while we used only current SIGN guidelines. This must be considered also as a potential limitation.

In summary, we were able to show a high frequency of non-evidence-based recommendations in S3-guidelines of psychiatry and psychotherapy. Furthermore, we could demonstrate that the size of the involved group seems to affect and promote the development of such recommendations. As guidelines should be practical tools to simplify the clinical decision-making processes based on scientific evidence in times of multiple treatment options, our findings support a view that new strategies to deal with clinically relevant questions where no evidence is available are needed. For reasons of practical applicability, guideline developers in general should be careful about the included amount of recommendations and the scientific evidence on which the recommendations are based.

## Data Availability

All data generated or analysed during this study are included in this published article. The raw data can be requested from the corresponding author.

## Abbreviations

AGREE: Appraisal of the Guidelines for Research and Evaluation instrument
AWMF: Association of the Scientific Medical Societies
CCP: Clinical consensus point
DELBI: German Rating Instrument for Guideline Valuation
DGN: The German association for Neurology
DGPPN: German Society for Psychiatry and Psychotherapy
GCP: Good Clinical Practice
GRADE: Grading of Recommendations Assessment, Development and Evaluation
NICE: National Institute of Clinical Excellence
SD: Standard deviation
SIGN: Scottish Intercollegiate Guidelines Network
SOP: Standard operating procedure
